# A 4-month-old baby boy presenting with anaphylaxis to a banana: a case report

**DOI:** 10.1186/1752-1947-8-62

**Published:** 2014-02-19

**Authors:** Andrew W O’Keefe, Moshe Ben-Shoshan

**Affiliations:** 1 Division of Allergy and Clinical Immunology, Room C-510, Montreal Children’s Hospital, 2300 rue Tupper, Montreal, QC H3H 1P3, Canada

## Abstract

**Introduction:**

Food allergy is the most common cause of anaphylaxis in children and recent studies suggest increased prevalence of both food allergy and anaphylaxis. Among foods, fruits are rarely implicated as the cause of anaphylaxis. Furthermore, anaphylaxis to fruit in the first months of life is rare. Although banana allergy has been well described in adults, there are only two case reports of anaphylaxis to banana in children.

**Case presentation:**

A 4-month-old Hispanic baby boy with a history of eczema presented to our emergency room with vomiting, urticaria and cyanosis following first exposure to a banana. He improved with administration of intramuscular epinephrine. Skin prick tests showed positive results for both fresh banana (4mm wheal/15mm erythema) and banana extract (8mm wheal/20mm erythema).

**Conclusions:**

Banana is not considered a highly allergenic food. However, as food allergy becomes more common and solid foods are being introduced earlier in babies, banana may become an important allergen to consider in cases of babies presenting with anaphylaxis.

## Introduction

Food allergy is the most common cause of anaphylaxis in children, with the usual foods implicated being: peanut, tree nuts, shellfish and milk [1]. Recent studies suggest an increase in the prevalence of both food allergies and anaphylaxis [2]. Although fruit allergy causing anaphylaxis has been well described in the literature, it is far rarer than the aforementioned foods. Among fruits, banana is a rare cause of anaphylaxis in babies. In a study comparing 40 children with reported banana allergy to 40 control patients with a history of atopic disease, only three of the 40 patients with self-reported banana allergy had a positive skin prick test using fresh banana and extract. None of the control subjects had a positive skin prick test to either fresh banana or banana extract. Of the remaining 37 patients, 20 consented to oral challenge with banana, all of which were negative. There was 100% correlation of skin prick testing with fresh banana and extract. All studied patients had detectable specific immunoglobulin E (IgE) to banana, regardless of history of reaction [3].

There have been several other case reports of allergic reaction to banana in babies ranging from 5 to 7 months of age. Three cases involved generalised urticaria only [4-6], whereas two reported anaphylaxis [4,7]. Three of the cases were confirmed with positive skin prick tests, whereas the remaining two were confirmed with specific IgE for banana.

In the adult population banana allergy is well documented. It is often associated with latex allergy (fruit-latex syndrome).

## Case presentation

**Figure 1:**
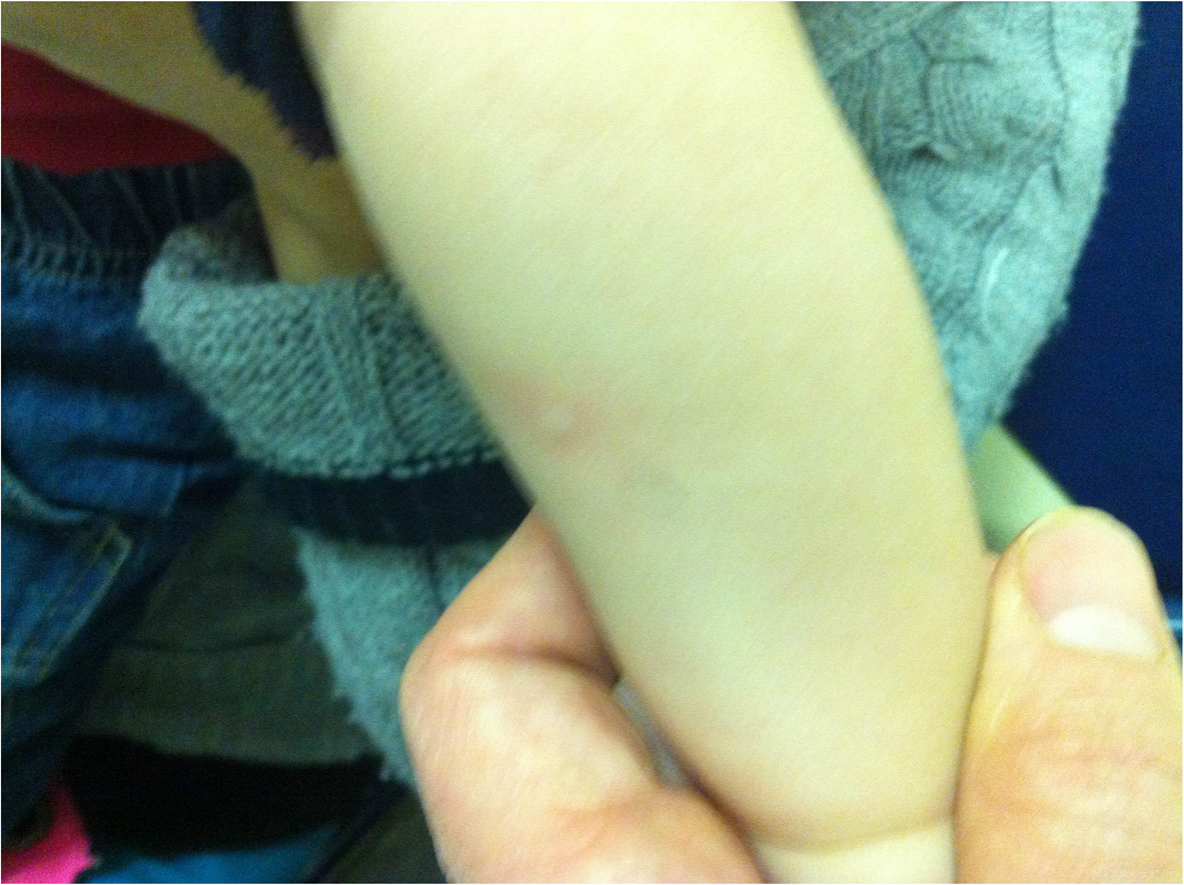
On presentation to the emergency room, the patient had diffuse urticaria and erythema.

A 4-month-old Hispanic baby boy with a history of eczema presented to our emergency room with vomiting, urticaria and cyanosis within 5 minutes of eating banana. This was his first known exposure to banana. On emergency room triage, he was tachycardic at 190 beats per minute, but his other vital signs were stable (temperature 36.9°C, respiratory rate 50 breaths/minute, saturation level of oxygen in haemoglobin 98% in room air). A physical examination was significant for diffuse urticaria and moderate perioral cyanosis. In our emergency room he was treated with intramuscular epinephrine (0.1mg, intramuscular, one dose) and oral diphenhydramine (6.25mg, one dose) with gradual improvement. He remained stable during a 6-hour observation period following the administration of epinephrine and was discharged home with a prescription for injectable epinephrine and out-patient consultation to an allergist (Figure 1).

He had a follow-up visit in the allergy clinic 6 months after his initial reaction. He had a positive skin test to banana extract with an 8mm wheal and 20mm of erythema. In addition, a skin prick test to fresh banana was positive with a 4mm wheal and 15mm erythema. There was no family history of atopy.

He continues to avoid banana in his diet and has had no further reactions.

## Discussion

Sensitisation to food antigens has been thought to occur through multiples routes including cutaneous, respiratory tract, and via the gastrointestinal tract through direct exposure or in breast milk. In this case, prior exposure to banana through the oral route has not been reported; however, sensitisation may have been established via other routes. Given that the patient has eczema and that impairment in skin barrier and exposure through the cutaneous route has been implicated in the pathogenesis of other food allergies [8-10], this might have contributed to prior sensitisation. Other possibilities include inadvertent oral exposure, or exposure through breast milk. Given that some of the major banana allergens may be heat sensitive [11] it is possible that exposure to fresh banana may have contributed to the severity of his symptoms.

## Conclusions

Anaphylaxis to banana in babies is rare, with only two cases being previously reported. We describe the youngest case of anaphylaxis to banana in a 4-month-old baby boy. Skin prick tests using fresh banana and commercial banana extract can be used to establish the diagnosis of banana allergy. As food allergy and anaphylaxis become more common, it will be important to consider banana as a cause of anaphylaxis in babies as this raw, unprocessed food is often introduced early. Prompt treatment with epinephrine and elimination diets are required to appropriately manage these life-threatening reactions.

## Consent

Written informed consent was obtained from the patient’s legal guardian(s) for publication of this case report and any accompanying images. A copy of the written consent is available for review by the Editor-in-Chief of this journal.

## Competing interests

The authors declare that they have no competing interests.

## Authors’ contributions

AOK performed the literature review and wrote the first draft of the manuscript. MBS collected the patient data and edited the manuscript. Both authors read and approved the final manuscript.
